# Analysis of clinical characteristics of ocular trauma patients at a single-center hospital in Northwest China

**DOI:** 10.3389/fmed.2026.1856156

**Published:** 2026-09-25

**Authors:** Tao Chen, Huping Song, Houxue Xia

**Affiliations:** Department of Ophthalmology, Xi'an People's Hospital (Xi'an Fourth Hospital), Xi'an, Shaanxi, China

**Keywords:** epidemiology, injury prevention, ocular trauma, pediatric injuries, rural eye health, seasonal patterns

## Abstract

**Purpose:**

To analyze the demographic characteristics, injury types, causes, and visual prognosis of ocular trauma patients in Northwest China, and to identify high-risk populations and seasonal patterns to inform targeted prevention strategies.

**Methods:**

A retrospective study was conducted at Xi'an People's Hospital in Shaanxi Province from January 2018 to June 2022. Data were collected on demographic characteristics, types of ocular trauma, causes of injury, injury sites, and visual acuity outcomes. Statistical analyses included chi-square tests, t-tests, and multivariable logistic regression to identify independent prognostic factors.

**Results:**

Among 1,671 hospitalized patients, 1,352 (80.9%) were male and 319 (19.1%) were female, with a mean age of 41.2 ± 19.8 years. The majority of patients were manual laborers (1,253, 75.0%) and resided in rural areas (1,244, 74.4%). Open-globe injuries accounted for 75.9% (*n* = 1,268) of cases, with rupture being the most common subtype (536, 32.1%). The most frequent injury site was the home (541, 32.4%), followed by the workplace (449, 26.9%). Sharp objects and blunt instruments were the leading causes of injury (1,181, 70.7%). Among 260 pediatric patients (≤18 years), 82.3% (*n* = 214) sustained open-globe injuries, predominantly caused by sharp household objects (135, 51.9%). Firework-related injuries showed a distinct seasonal peak in February (50%, corresponding to the Lunar New Year). Multivariable analysis identified increasing age (per year, OR = 0.972, 95% CI: 0.961–0.982, *p* < 0.001), Zone III injury (compared to Zone I, OR = 0.421, 95% CI: 0.243–0.729, *p* = 0.002), and open-globe injury (OR = 0.096, 95% CI: 0.057–0.160, *p* < 0.001) as independent negative prognostic factors for achieving final visual acuity ≥0.5.

**Conclusion:**

Ocular trauma in Northwest China predominantly affects male manual laborers in rural areas. Open-globe injuries, particularly in children, carry a poor prognosis. Seasonal peaks during the Lunar New Year highlight opportunities for targeted public health interventions. These findings underscore the need for region-specific prevention strategies, including child safety education, workplace protection regulations, and holiday safety campaigns.

## 1 Introduction

Ocular trauma remains a leading cause of preventable vision loss worldwide. Approximately 1.6 million people are blind due to ocular trauma, and it is a primary cause of monocular blindness in China (1, 2). The condition severely impacts patients’ work capacity and quality of life, while imposing a substantial economic burden on families and society (3). Over 90% of ocular traumas are preventable in clinical settings (4). The epidemiological characteristics of ocular trauma vary significantly across regions due to differences in socioeconomic conditions, occupational structures, and environmental factors (5). Understanding these regional variations is essential for developing targeted prevention strategies and optimizing clinical management. This study was conducted in Shaanxi Province, a representative region of Northwest China characterized by its mixed urban-rural population, extensive agricultural activities, and growing industrial sectors. Unlike the densely populated coastal cities such as Beijing or Dalian studied in prior research, Shaanxi's economy relies heavily on manufacturing, mining, and agriculture, which inherently involve high-risk occupations (6). Notably, rural areas in Shaanxi account for over 50% of the population, with a substantial proportion engaged in manual labor—factors that may elevate ocular trauma risks compared to more urbanized regions (7).

While previous studies, including a 2022 epidemiological analysis of ocular trauma in Shaanxi and surrounding areas, have provided foundational insights (8), this study offers updated data spanning January 2018 to June 2022, capturing post-pandemic trends and expanding on granular variables such as injury zoning, pediatric trauma characteristics, and rural-urban disparities. Furthermore, our analysis uniquely integrates occupational classifications (manual vs. intellectual laborers) and correlates injury mechanisms with visual prognosis—aspects that have been underexplored in previous regional studies, which primarily focused on basic demographic trends without detailed zonal or pediatric subgroup analyses (8, 9).

Therefore, the objective of this study is to provide a comprehensive analysis of the clinical characteristics of ocular trauma patients at a single tertiary center in Northwest China. Specifically, we aim to: (1) describe the demographic and epidemiological profile of these patients, (2) identify key injury patterns and seasonal trends, and (3) determine independent prognostic factors for visual outcomes. By focusing on a high-risk population in an understudied geographic context, this work addresses gaps in localized prevention strategies and underscores the urgency of labor protection reforms in Northwest China.

## 2 Material and methods

### 2.1 Study design and participants

This retrospective study collected and organized the data of patients with ocular trauma admitted to Xi'an People's Hospital in Shaanxi Province from January 2018 to June 2022. The diagnostic criteria referred to the international disease classification standards for ocular trauma diagnosis (5, 6)^.^

Inclusion criteria: patients with ocular trauma admitted to a single-center hospital in northwest China from January 2018 to June 2022.

#### Exclusion criteria

(1) patients with a history of severe pre-existing ocular diseases (e.g., glaucoma, diabetic retinopathy, advanced cataracts) that could confound the assessment of visual acuity; (2) patients whose ocular trauma was a secondary complication of life-threatening systemic events (e.g., stroke) where the primary treatment focus was not the eye; and (3) patients who received treatment solely in the outpatient department. Patients with ocular injuries accompanied by non-life-threatening systemic involvement were not excluded.

The decision to include only hospitalized patients was made to ensure the availability of complete medical records, including detailed diagnostic workup, surgical notes, and pre- and postoperative visual acuity measurements, which are critical for the study's objectives. As a referral center, our hospital treats the most severe cases from across the region.

For the purpose of this study, pediatric patients were defined as individuals aged 18 years and younger.

### 2.2 Data collection

Data from 1,671 cases included age, gender, occupation, place of residence, type of settlement, time and cause of injury, injury location, time of consultation, length of hospital stay, classification and distribution of ocular trauma, preoperative and postoperative visual acuity (VA), accompanying symptoms, and total hospitalization cost.

### 2.3 Trauma classification

The diagnosis of ocular trauma was based on a comprehensive clinical examination by an ophthalmologist. The International Classification of Diseases, Tenth Revision (ICD-10) codes for ocular trauma (S05.-) were used to identify eligible cases from the hospital's database (10).

Injuries were classified as either mechanical or non-mechanical based on the Birmingham Eye Trauma Terminology (BETT) system (11). Mechanical injuries were further classified as closed-globe or open-globe injuries.

**Open-globe injuries (OGIs)** were sub-classified as:

- Rupture: Full-thickness wound of the eyewall caused by blunt force
- Penetrating: Single full-thickness entry wound from a sharp object
- Perforating: Through-and-through wound with both entry and exit sites
- Intraocular foreign body (IOFB): Retention of a foreign body within the globe

**Closed-globe injuries (CGIs)** were classified as:

- Contusion: Injury from blunt force without full-thickness wound
- Lamellar laceration: Partial-thickness wound of the eyewall

Non-mechanical injuries included chemical, thermal, and radiation burns.

The anatomical zone of injury for OGIs was also recorded according to BETT standards (11):

- Zone I: Injury confined to the cornea and corneal limbus
- Zone II: Injury involving the sclera up to 5 mm posterior to the limbus
- Zone III: Injury involving the sclera more than 5 mm posterior to the limbus

### 2.4 Visual acuity assessment

Visual acuity (VA) was measured using a standard Snellen chart at a distance of 6 meters. For the purposes of analysis, Snellen fractions were converted to decimal visual acuity values using standard conversion tables (e.g., 6/6 = 1.0, 6/12 = 0.5, 6/60 = 0.1). These decimal values were then categorized into the 5-grade scale described by Wang et al. (9) (Table 1). Grade 1 (≥ 0.5) corresponds to Snellen acuity of 6/12 or better, which served as the threshold for good visual prognosis in our regression analysis.

**Table 1:** Five-grade classification of visual acuity used in the study.

Grade	Visual Acuity
1	≥ 0.5
2	0.2–0.4
3	0.025–0.19
4	Light perception (LP)—0.02
5	No light perception (NLP)

**‘Final VA’** was defined as the best-corrected visual acuity measured at the time of discharge from the hospital. Post-discharge follow-up data were not consistently available for all patients and were therefore not included in this analysis.

### 2.5 Statistical analysis

Statistical analyses were performed using SPSS software (version 27.0; IBM Corp., Armonk, NY, USA). Continuous variables were tested for normality using the Shapiro–Wilk test. Normally distributed data are presented as mean ± standard deviation (SD), and non-normally distributed data as median with interquartile range (IQR). Categorical data were expressed as frequencies and percentages (n, %).

For comparisons between groups, the following tests were used:

- Independent sample t-test or Mann–Whitney U test for continuous variables
- One-way ANOVA or Kruskal–Wallis test for continuous variables across multiple groups
- Chi-square (*χ*^2^) test for categorical variables

To identify independent prognostic factors for visual outcome (final VA ≥ 0.5 vs. < 0.5), a binary logistic regression analysis was performed, adjusting for potential confounders including age, sex, injury type (open vs. closed globe), injury zone, and consultation delay. Results are reported as odds ratios (OR) with 95% confidence intervals (CI). A *p*-value < 0.05 was considered statistically significant.

## 3 Results

### 3.1 Demographic characteristics

Among the 1,671 cases, there were 1,352 males (80.9%) and 319 females (19.1%), with a male-to-female ratio of 4.2:1. The average age was 41.2 ± 19.8 years, ranging from 1 to 89 years old. There was no significant difference in age between genders (*P* > 0.05), with an average age of 41.5 ± 18.6 years for males and 40.3 ± 24.2 years for females. Age distribution showed that the peak proportion of ocular trauma occurred in the 41–50-year-old group (21.1%, *n* = 352), followed by the 51–60-year-old group (19.4%, *n* = 324) and the 31–40-year-old group (15.8%, *n* = 264). The highest proportion of ocular trauma in females (22.5%, *n* = 72) was in the 1–10-year-old age group (Figure 1). The pediatric group (≤18 years) comprised 260 patients (15.6%), with a male-to-female ratio of 1.9:1 (171 males, 65.8%; 89 females, 34.2%).

**Figure 1:**
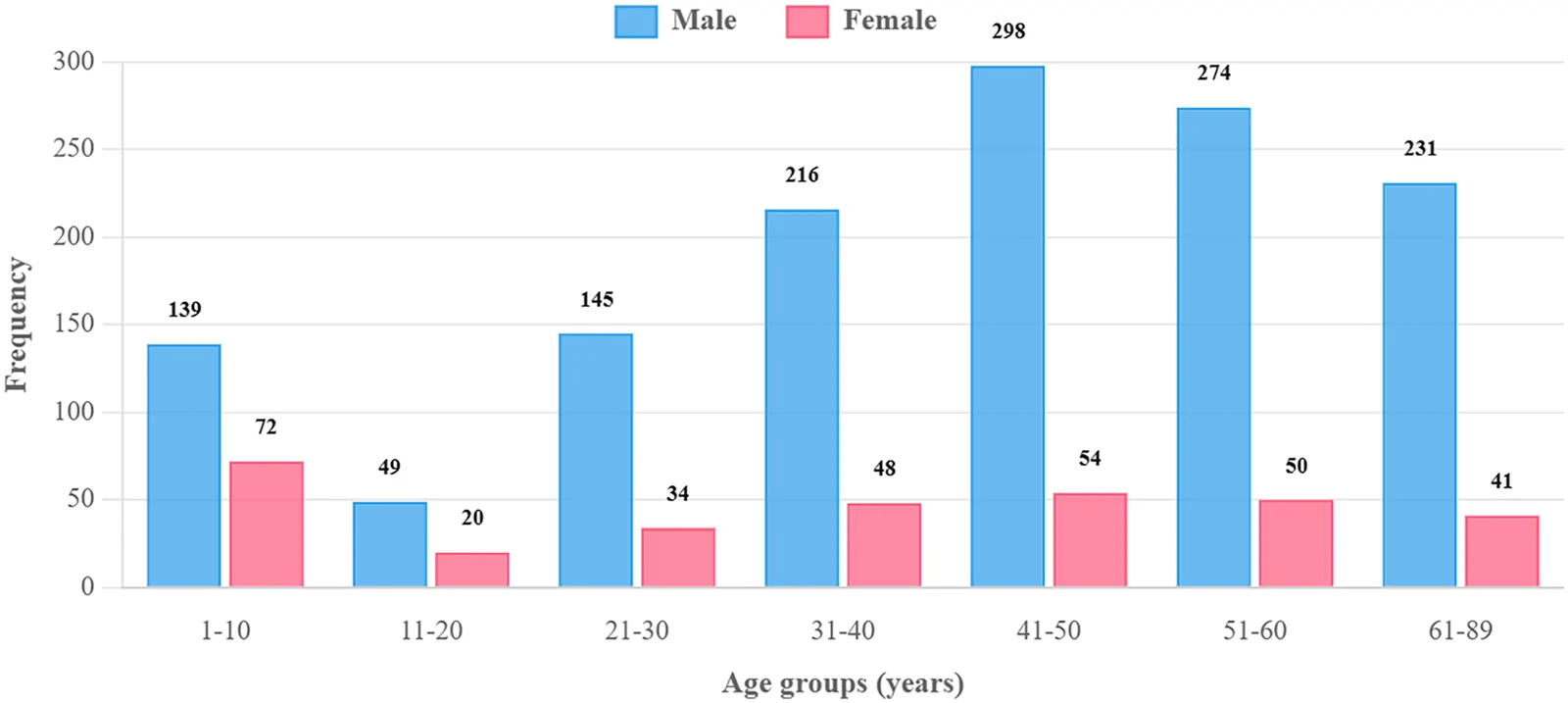
Age and gender distribution of ocular trauma patients (*n* = 1,671). The peak incidence occurred in the 41-50-year-old age group (21.1%). Among females, the highest proportion of injuries occurred in the 1−10-year-old age group (22.5% of all female patients). Male:female ratio was 4.2:1 overall.

Among the 1,671 patients, the most common occupation was manual laborer (75.0%, *n* = 1,253), followed by intellectual laborer (25.0%, *n* = 418). The incidence of ocular trauma in rural areas (74.4%, *n* = 1,244) was higher than in urban areas (25.6%, *n* = 427, *P* < 0.001). There was no statistically significant difference in the incidence of right and left eye injuries, with 850 cases (50.9%) in the right eye and 809 cases (48.4%) in the left eye. Twelve cases (0.7%) had bilateral eye injuries. The delay in consultation for patients arriving at the consultation room was also assessed. As shown in Figure 2, 338 cases (20.2%) arrived at the consultation room for treatment within 6 h, 505 cases (30.2%) arrived within 6–12 h, and 348 cases (20.8%) arrived within 12–24 h after injury. Among patients with rupture and penetrating injuries, 92.3% (*n* = 904) presented to the hospital within 3 days of the injury. Patients who presented more than one month after the injury (*n* = 8, 0.5%) were most frequently diagnosed with blunt contusions (*n* = 5, 62.5%). The mean hospital stay for all patients was 6.65 ± 4.27 days. However, the median hospital stay for patients with globe rupture (8 days, IQR 5–11) was significantly longer than for those with corneal penetration (6 days, IQR 4–8, *p* < 0.05).

**Figure 2:**
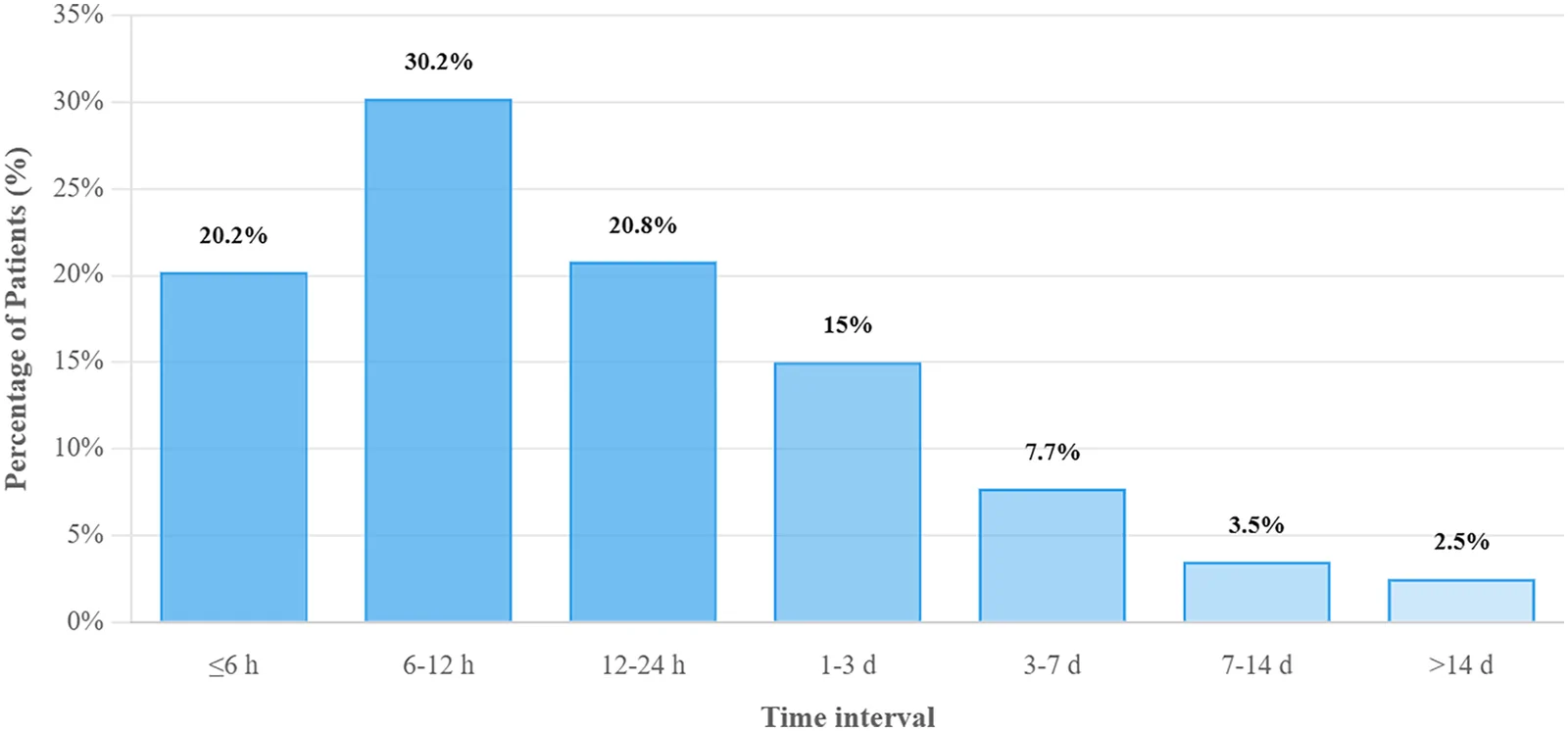
Time interval between injury and presentation to hospital (*n* = 1,671). The majority of patients (71.2%) presented within 24 h. Patients with penetrating and rupture injuries presented earlier, with 92.3% arriving within 3 days.

### 3.2 Types and causes of injury

#### 3.2.1 Classification and zoning of ocular trauma patients

As shown in Table 2, the proportion of open globe injuries (1,268 cases, 75.9%) was higher than that of closed injuries (295 cases, 17.7%), adnexal injuries (103 cases, 6.2%), and non-mechanical injuries (5 cases, 0.3%). Among the open globe injuries, rupture was the most common (536 cases, 32.1%), followed by perforation (443 cases, 25.5%) and intraocular foreign bodies (254 cases, 15.2%). In the 1,268 cases of open globe injuries, Zone I was the most common site of injury distribution (599 cases, 47.2%), followed by Zone II (374 cases, 29.5%) and Zone III (295 cases, 23.3%). At the same time, among rupture injuries (*n* = 536), the most common site was Zone II (228 cases, 42.6%); among perforating injuries (*n* = 443), the most common site was Zone I (326 cases, 73.6%).

**Table 2:** Demographics and clinical characteristics of patients hospitalized for ocular trauma at a tertiary hospital in northwest China (*n* = 1,671).

Characteristic	*n* (%)	*p*-value
Total cases		
Total	1,671	
Gender		
Male	1,352 (80.9)	<0.001
Female	319 (19.1)	
Age groups (years)		
1–10	211 (12.6)	<0.001
11–20	69 (4.1)	
21–30	179 (10.7)	
31–40	264 (15.8)	
41–50	352 (21.1)	
51–60	324 (19.4)	
61–89	272 (16.3)	
Occupation		
Manual laborer	1,253 (75.0)	<0.001
Intellectual laborer	418 (25.0)	
Location		
Rural	1,244 (74.4)	<0.001
Urban	427 (25.6)	
Injury site		
Home	541 (32.4)	<0.001
Workplace	449 (26.9)	
Agricultural site	309 (18.5)	
Public place	293 (17.5)	
School	79 (4.7)	
Laterality		
Right eye (OD)	850 (50.9)	0.094
Left eye (OS)	809 (48.4)	
Bilateral (OU)	12 (0.7)	
Type of ocular trauma		
Open-globe injury	1,268 (75.9)	<0.001
Rupture	536 (32.1)	
Perforating	443 (26.5)	
Intraocular foreign body	254 (15.2)	
Penetrating	20 (1.2)	
Through-and-through	15 (0.9)	
Closed-globe injury	295 (17.7)	
Blunt contusion	225 (13.5)	
Lamellar laceration	14 (0.8)	
Mixed types	31 (1.9)	
Superficial foreign body	25 (1.5)	
Non-mechanical injury	5 (0.3)	
Ocular adnexal injury	103 (6.2)	
Pediatric subgroup (≤18 years)		
Total	260 (15.6)	
Male	171 (65.8)	0.003
Female	89 (34.2)	
Open-globe injury	214 (82.3)	<0.001
Most common site: Home	156 (60.0)	<0.001
Most common cause: Sharp objects	135 (51.9)	<0.001

#### 3.2.2 Distribution of injury sites for ocular trauma patients

The most common site of ocular trauma occurrence was the home (541 cases, 32.4%), followed by the workplace(e.g., Office, factory) (449 cases, 26.9%), agricultural sites (309 cases, 18.5%), public places (293 cases, 17.5%), and schools (79 cases, 4.7%). Perforating injuries often occurred in homes, workplaces, and agricultural sites, while contusion and rupture injuries frequently occurred in homes, public places, and workplaces. Intraocular foreign bodies were more common in workplaces and agricultural sites. See Table 3 for details on the distribution of ocular trauma types at the sites of occurrence for hospitalized patients.

**Table 3:** Distribution of ocular trauma types by injury site (*n* = 1,671).

Type of Ocular Trauma	Workplace *n* (%)	Public Places *n* (%)	Agricultural Sites *n* (%)	Schools *n* (%)	Home *n* (%)	Total
Perforating	106 (23.9)	28 (6.3)	94 (21.2)	44 (9.9)	171 (38.6)	443
Penetrating	3 (20.0)	1 (6.7)	0 (0.0)	4 (26.7)	7 (46.7)	15
Through-and-Through	12 (60.0)	1 (5.0)	2 (10.0)	1 (5.0)	4 (20.0)	20
Blunt Contusion	41 (18.2)	59 (26.2)	39 (17.3)	6 (2.7)	80 (35.6)	225
Rupture	124 (23.0)	143 (26.6)	65 (12.1)	14 (2.6)	192 (35.7)	538
Intraocular Foreign Body	125 (43.1)	19 (6.6)	89 (30.7)	5 (1.7)	52 (17.9)	290
Lamellar Laceration	13 (34.2)	4 (10.5)	9 (23.7)	1 (2.6)	11 (28.9)	38
Non-mechanical Injury	2 (40.0)	1 (20.0)	0 (0.0)	0 (0.0)	2 (40.0)	5
Adnexal Injury	23 (23.7)	37 (38.1)	11 (11.3)	4 (4.1)	22 (22.7)	97
Total	449 (26.9)	293 (17.5)	309 (18.5)	79 (4.7)	541 (32.4)	1,671

Chi-square test for overall comparison of injury type distribution across sites, *p* < 0.001.

#### 3.2.3 Distribution of causes of injury for ocular trauma patients

Among all cases, the most common cause of injury was blunt instruments, sharp objects, or metal splashes (1,181 cases, 70.7%), with the most common being occupational injuries occurring at the workplace (418 cases, 35.4%), followed by eye injuries caused by blunt and sharp objects in the home (330 cases, 27.9%) (Table 4).

**Table 4:** Distribution of injury causes by injury site (*n* = 1,671).

Cause	Home *n* (%)	Workplace *n* (%)	Agricultural Site *n* (%)	Public Place *n* (%)	School *n* (%)	Total
Sharp/Blunt/Metal	330 (27.9)	418 (35.4)	239 (20.2)	138 (11.7)	56 (4.7)	1,181
Falls	66 (40.7)	12 (7.4)	23 (14.2)	54 (33.3)	7 (4.3)	162
Plant-related	15 (10.6)	12 (8.5)	80 (56.7)	31 (22.0)	3 (2.1)	141
Traffic Accident	1 (1.3)	0 (0.0)	2 (2.6)	73 (96.1)	0 (0.0)	76
Fireworks	50 (67.6)	0 (0.0)	0 (0.0)	16 (21.6)	8 (10.8)	74
Other	79 (57.2)	7 (5.1)	0 (0.0)	27 (19.6)	5 (3.6)	118
Total	541	449	344	339	79	1,752*

*Total exceeds 1,671 due to patients with multiple causes. Chi-square test *p* < 0.001.

In addition to blunt instruments, sharp objects, or metal splashes, common causes of injury included falls (162 cases, 9.7%), plant injuries (141 cases, 8.4%), traffic accidents (76 cases, 4.5%), and fireworks (74 cases, 4.4%). Falls mainly occurred in homes (66 cases, 40.7%) and public places (54 cases, 33.3%), with the majority of eye injuries from falls being ruptures (91 cases, 56.2%). Traffic accidents all occurred in public places, with the main eye injuries being ruptures (35 cases, 46.1%). Plant injuries mainly occurred in agricultural sites (80 cases, 56.7%), with the main eye injuries being perforations (37 cases, 46.2%), intraocular foreign bodies (17 cases, 21.5%), and ruptures (16 cases, 20.0%). Firework injuries mainly occurred in homes (44 cases, 59.5%), with the majority of firework-related eye injuries being ruptures (36 cases, 48.6%), contusions (16 cases, 21.6%), and intraocular foreign bodies (10 cases, 13.5%). Notably, 50% of firework-related injuries occurred in February (Lunar New Year), significantly higher than in any other month (*χ*^2^ test, *p* < 0.001; Figure 3).

**Figure 3:**
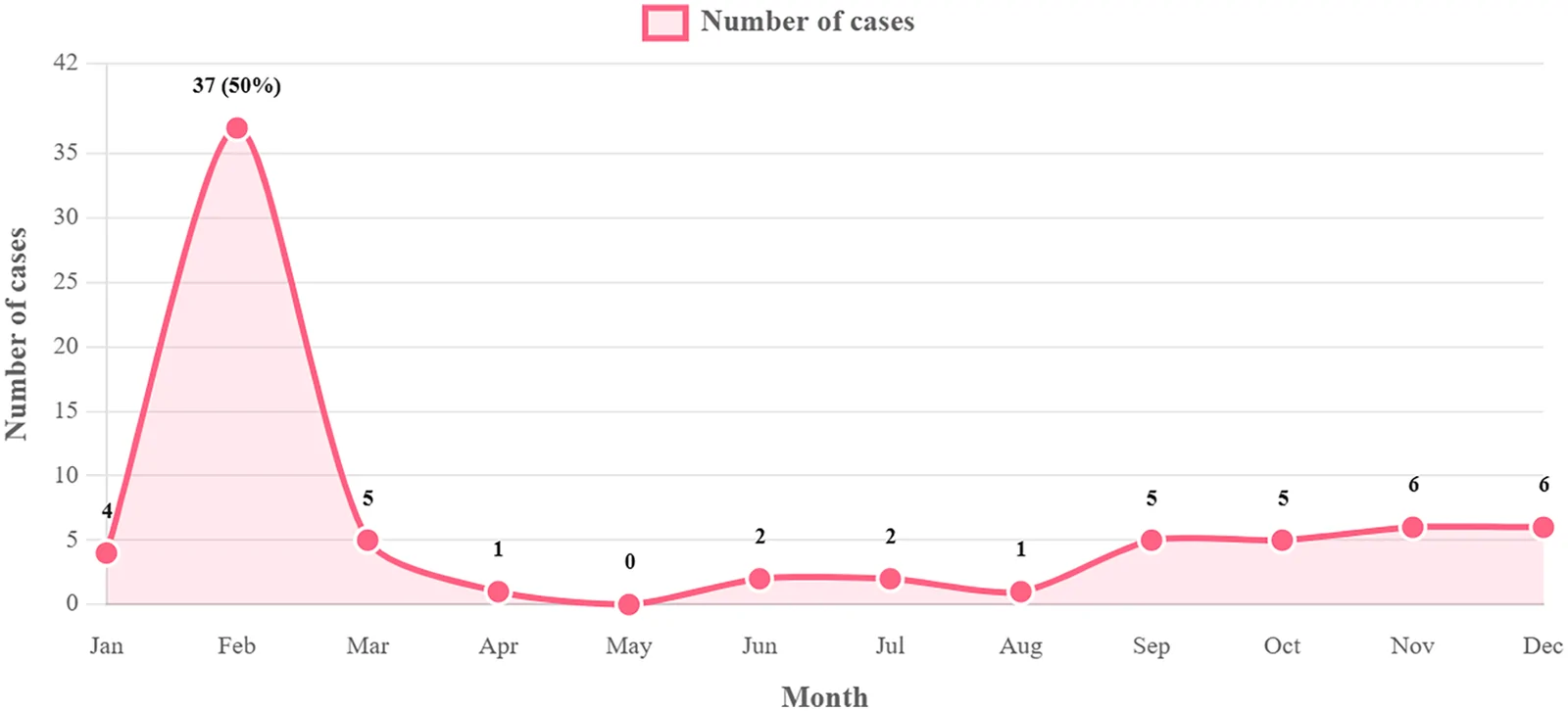
Monthly distribution of firework-related ocular trauma (*n* = 74). A distinct peak is observed in February (50% of all firework injuries), corresponding to the Lunar New Year celebrations. The proportion of firework injuries in February was significantly higher than in any other month (*p* < 0.001).

### 3.3 Pediatric ocular trauma characteristics

A total of 260 patients were identified in the pediatric age group (under 18 years old). Within this group, 211 (81.2%) were aged 1–10 years and 49 (18.8%) were aged 11–18 years.

Among pediatric patients, open-globe injuries (OGI) were the most prevalent, accounting for 214 cases (82.3%). Perforating injuries (*n* = 134, 62.6%) were the most common subtype of OGI. The most frequent injury site was the home (*n* = 156, 60.0%), and the most common cause was sharp household objects (*n* = 135, 51.9%), followed by blunt objects (*n* = 40, 15.4%) and falls (*n* = 30, 11.5%).

In the pediatric group, the initial VA was predominantly Grade 4 (LP-0.02, *n* = 151, 58.1%). Postoperatively, 62 patients (23.8%) achieved VA Grade 2 or better, while 158 patients (60.8%) remained at Grade 4 or 5. The visual prognosis for pediatric patients with OGI was significantly worse than for those with closed-globe injuries (*p* < 0.001). Notably, the proportion of OGI in pediatric patients (82.3%) was higher than that in adult patients (74.7%), and the final visual outcomes appeared worse in children, although formal statistical testing was not conducted.

### 3.4 Initial visual acuity (VA) and prognostic VA

The distribution of preoperative and postoperative VA is shown in Table 5. After excluding 3 patients who could not cooperate with testing and 74 pediatric patients who could not understand the examination, a total of 1,594 patients were included in the VA analysis. Preoperatively, the majority of patients (916, 58%) had VA Grade 4 (LP to 0.02). Postoperatively, the proportion of patients with Grade 4 decreased to 839 (53%), while the proportion achieving Grade 2 or better increased from 16% to 25%. The number of patients with Grade 5 (NLP) decreased from 245 (15%) preoperatively to 127 (8%) postoperatively. Final VA was strongly correlated with initial VA (*r* = 0.764, *p* < 0.001).

**Table 5:** Comparison of preoperative and postoperative visual acuity (VA) in patients with ocular trauma (*n* = 1,594).

VA Grade	Visual Acuity	Preoperative *n* (%)	Postoperative *n* (%)
1	≥ 0.5	117 (7)	179 (11)
2	0.2–0.4	141 (9)	214 (14)
3	0.025–0.19	162 (10)	222 (14)
4	LP—0.02	916 (58)	839 (53)
5	NLP	245 (15)	127 (8)

VA was measured using a standard Snellen chart and converted to a 5-grade scale. Grade 1 = ≥0.5; Grade 2 = 0.2–0.4; Grade 3 = 0.025–0.19; Grade 4 = LP (light perception) to 0.02; Grade 5 = NLP (no light perception). Preoperative and postoperative VA were significantly correlated (Spearman's *r* = 0.764, *p* < 0.001).

### 3.5 Treatment status

Conservative treatment was performed in 64 cases (3.9%), while emergency surgery was performed in the remaining 1,607 cases (96.2%), including procedures such as globe rupture repair, vitreoretinal surgery, lens extraction, foreign body removal, and intraocular injection, etc. Four cases underwent enucleation due to severe globe rupture. 733 cases received phase II（Refers to systematic follow-up surgeries performed after acute-phase management of ocular trauma, aimed at optimizing functional or structural outcomes, and serves as a critical transitional phase in trauma care bridging ‘emergency intervention’ to ‘rehabilitation’."）surgery, accounting for 43.87%.

Patients with closed-globe injuries (CGI) had a significantly better visual prognosis than those with open-globe injuries (OGI). Specifically, 48% (*n* = 141) of CGI patients achieved a final VA of Grade 2 or better, compared to only 21% (*n* = 266) of OGI patients (*p* < 0.001). Conversely, poor outcomes (Grade 4 or 5) were observed in 62% (*n* = 787) of OGI patients, versus 35% (*n* = 103) of CGI patients. Figures 4a,b illustrate the shift in VA grade distribution from pre- to post-treatment for both groups.

**Figure 4:**
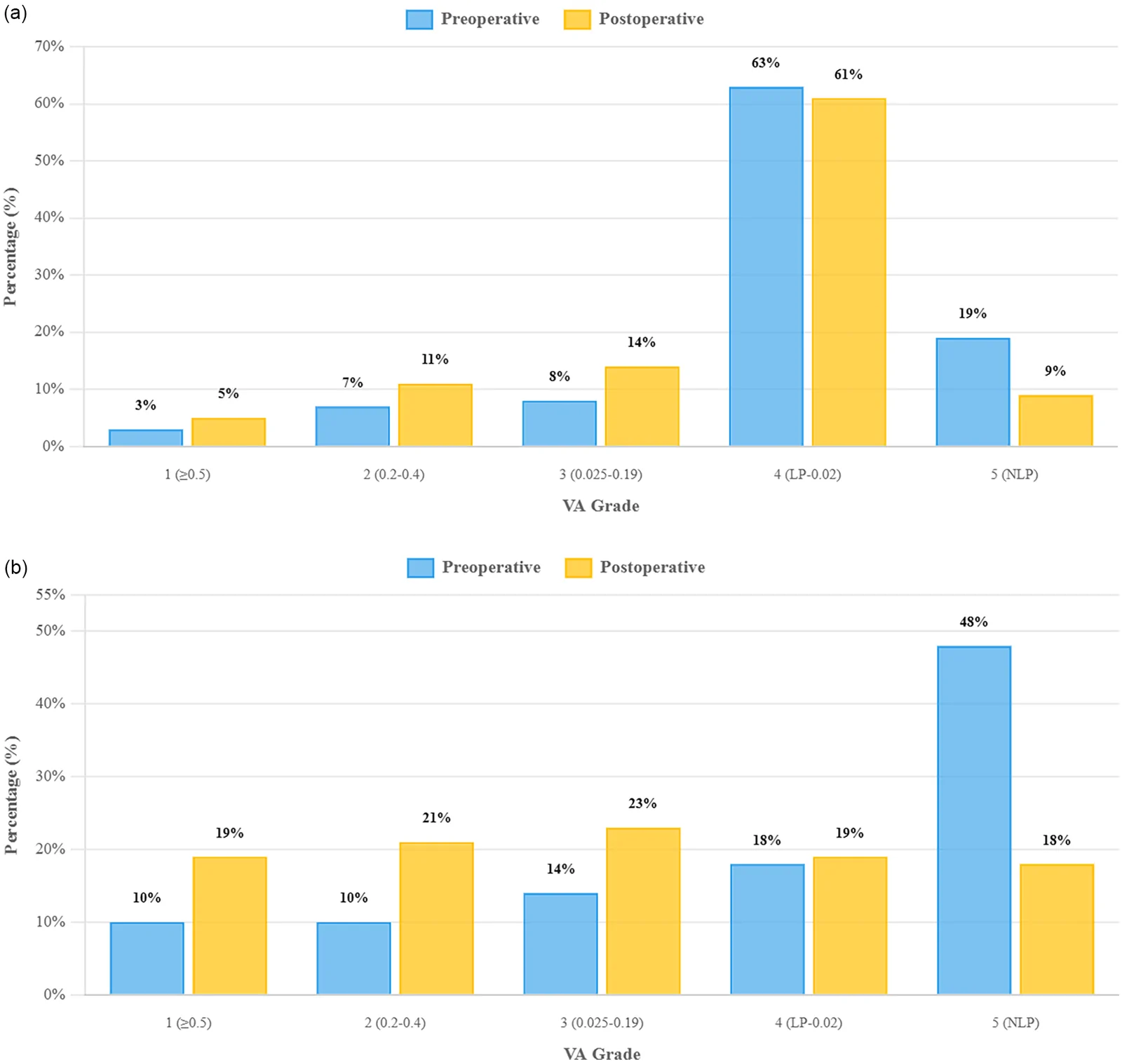
Comparison of preoperative and postoperative visual acuity (VA) distribution. **(a)** Patients with open-globe injuries (OGI, *n* = 1,268): The proportion of patients with VA Grade 4 (LP-0.02) remained high postoperatively (61% vs. 63% preoperatively), with only 21% achieving Grade 2 or better. **(b)** Patients with closed-globe injuries (CGI, *n* = 295): A greater proportion achieved Grade 2 or better postoperatively (48% vs. 19% preoperatively), demonstrating significantly better visual prognosis compared to OGI patients (*p* < 0.001). VA grading: Grade 1 (≥0.5), Grade 2 (0.2−0.4), Grade 3 (0.025−0.19), Grade 4 (LP-0.02), Grade 5 (NLP).

Among open-globe injuries, there was a moderate correlation between final VA and injury zone (Spearman's *r* = 0.211, *p* < 0.001). Patients with Zone I injuries had significantly better visual prognosis than those in Zone II or Zone III (*r* = 0.244, *p* < 0.001).

Multivariable analysis: After adjusting for potential confounders(including age, sex, injury type, injury zone, and consultation delay), multivariable logistic regression revealed that increasing age (per year, OR = 0.972, 95% CI: 0.961-0.982, *p* < 0.001), Zone III injury (compared to Zone I, OR = 0.421, 95% CI: 0.243-0.729, *p* = 0.002), and open-globe injury (OR = 0.096, 95% CI: 0.057-0.160, *p* < 0.001)</ins > were independent negative prognostic factors for achieving a final VA ≥ 0.5. The results are summarized in Table 6.

**Table 6:** Multivariable logistic regression analysis of factors associated with final visual acuity ≥ 0.5.

Variable	Reference Category	Adjusted OR	95% CI	*p*-value
Age (per year increase)	—	0.972	0.961–0.982	<0.001
Zone III injury	zone I	0.421	0.243–0.729	0.002
Open-globe injury	closed-globe injury	0.096	0.057–0.160	<0.001

OR < 1 indicates a negative prognostic factor for achieving final VA ≥ 0.5. Variables were adjusted for age, sex, injury type, injury zone, and consultation delay.

## 4 Discussion

This study provides a comprehensive epidemiological and clinical analysis of 1,671 hospitalized ocular trauma patients at a tertiary referral center in Northwest China. Our findings highlight the predominant burden among male manual laborers in rural areas, the high proportion of open-globe injuries, and the importance of injury zoning and timely presentation as key determinants of visual prognosis. These results have important implications for region-specific prevention strategies and clinical management.

### 4.1 Demographic patterns and regional comparisons

Our findings align with prior studies indicating male predominance and occupational hazards as key risk factors for ocular trauma (8, 9, 12). The male-to-female ratio of 4.2:1 in our cohort is consistent with reports from other regions of China, including central China (3.2:1) (9) and Dalian (3.5:1) (12). The predominance of manual laborers (75.0%) in our study is substantially higher than that reported in coastal cities, reflecting the different occupational structure of Northwest China.

Rural ocular trauma incidence in Shaanxi (74.4%) far exceeds reported rates from Dalian (approximately 40%) (12), likely due to Shaanxi's larger rural workforce engaged in unprotected agricultural and industrial activities. A 2022 Shaanxi study (8) highlighted similar demographic trends but did not analyze injury zoning or pediatric cases. Our study extends these findings by demonstrating that workplace injuries in Shaanxi (26.9%) were more prevalent than in Beijing-based studies (∼15%), reflecting differences in industrial safety regulations and enforcement.

### 4.2 Injury patterns and occupational risk factors

The predominance of open-globe injuries (75.9%) in our cohort is notably higher than in other Chinese studies (typically 40%–60%) (9, 12). This likely reflects our hospital's role as a tertiary referral center that receives the most severe cases, as well as the high-risk occupational environment in the region. The majority of open-globe injuries were caused by sharp objects, blunt instruments, and metal splashes—injuries commonly associated with agricultural work, manufacturing, and construction.

Among manual laborers (*n* = 1,253), injury sites were distributed as follows: workplace 33.3% (*n* = 417), agricultural sites 24.0% (*n* = 300), residential homes 25.8% (*n* = 323), and public places 16.9% (*n* = 212). These data indicate that ocular trauma in manual laborers occurs not only at formal workplaces but also during home-based labor activities such as installation, renovation, and maintenance. In contrast, among intellectual laborers (*n* = 417), injuries occurred predominantly in residential homes (52.3%, *n* = 218), followed by public places (19.4%, *n* = 81) and schools (18.7%, *n* = 78), while workplace injuries accounted for only 7.4% (*n* = 31). The lower incidence of workplace injuries among intellectual laborers likely reflects the safer nature of office environments.

The urban-rural disparity in injury sites is noteworthy. In urban areas, the main injury sites were workplaces (37.9%), residential homes (29.5%), and public places (24.4%). In rural areas, residential homes (33.4%), agricultural sites (24.6%), and workplaces (23.1%) were the predominant locations. Several factors contribute to the higher rural injury rates: (1) a higher proportion of left-behind elderly and children without adequate supervision; (2) inadequate infrastructure and less stringent implementation of occupational safety measures in rural enterprises; and (3) less rigorous road safety management and traffic control.

### 4.3 Pediatric ocular trauma

Pediatric ocular trauma represents a particularly concerning subgroup in our study. Among 260 pediatric patients, 82.3% sustained open-globe injuries, predominantly caused by sharp household objects (51.9%) and occurring at home (60.0%). This finding is consistent with studies from other developing regions where unsupervised children playing with sharp objects at home are at high risk (13).

These descriptive findings suggest a poorer prognosis in this group—with 60.8% remaining at VA Grade 4 or 5 post-treatment—underscore the need for targeted child safety education in rural households. Although no formal statistical comparison was performed, the higher proportion of open-globe injuries in children likely contributes to this observation. Parents and caregivers should be educated about the dangers of sharp objects and the importance of safe storage. Schools should strengthen safety education, particularly regarding protective measures during sports activities. Notably, studies have shown that badminton accounts for a high proportion (60.7%) of sports-related eye injuries (14), highlighting the need for protective eyewear during high-risk activities.

### 4.4 Injury zones and prognosis

A key contribution of this study is the correlation between injury zone and visual prognosis. Patients with Zone I injuries had significantly better outcomes than those with Zone II or Zone III injuries. This finding is consistent with the known anatomical vulnerability of the posterior segment and the greater complexity of surgical intervention required for Zone III injuries (15). The prognostic significance of injury zoning supports its routine use in clinical assessment and outcome prediction.

Our finding that initial VA was strongly correlated with final VA (*r* = 0.764, *p* < 0.001) is consistent with previous studies (9, 16), reinforcing the prognostic value of initial VA as a rapid clinical indicator.

### 4.5 Seasonal patterns and prevention opportunities

A distinctive finding of this study is the seasonal peak of firework-related injuries during the Lunar New Year (February), accounting for 50% of all firework injuries. This pattern is consistent with previous Chinese studies (17). The COVID-19 pandemic provided a natural experiment: during the Spring Festival of 2020 and 2021, only one case of firework-related injury was recorded, reflecting the effectiveness of social restrictions in reducing injury rates.

This seasonal pattern highlights a clear opportunity for public health intervention. Cities have successfully implemented fireworks restrictions, eliminating such injuries at the source. Our data suggest that rural areas would benefit from similar managed and secure viewing policies. Public awareness campaigns timed before the Lunar New Year could significantly reduce the burden of firework-related ocular trauma.

### 4.6 Treatment and outcomes

The majority of patients (96.2%) required emergency surgery, reflecting the severity of injuries in our cohort. The significant improvement in VA outcomes post-treatment—with the proportion achieving Grade 2 or better increasing from 16% to 25%—demonstrates the effectiveness of specialized ophthalmic surgical care.

The finding that delayed presentation >24 h was an independent negative prognostic factor underscores the importance of prompt medical attention. Prolonged time to consultation increases the risk of intraocular infection and preventable complications (18). Public education campaigns should emphasize the urgency of seeking immediate ophthalmic care for any eye injury.

### 4.7 Limitations

Several limitations of this study should be acknowledged. First, this is a single-center, hospital-based study. The inclusion of only hospitalized patients introduces a selection bias towards more severe ocular trauma. Patients with minor injuries treated in outpatient settings or at primary care clinics were not captured. Therefore, our findings, particularly the high proportion of open-globe injuries (75.9%), may not be representative of the full spectrum of ocular trauma in the community and should be generalized with caution.

Second, our analysis of final VA was based on measurements taken at discharge, and we lacked standardized long-term follow-up data. This limits our ability to assess the full recovery trajectory and the impact of delayed complications. Future studies should incorporate structured follow-up protocols.

Third, due to economic constraints, some patients may not have undergone secondary surgery, potentially affecting long-term visual outcomes. Follow-up was difficult to maintain, particularly for rural patients.

Fourth, our classification of injuries, while based on BETT standards, relied on retrospective chart review, which may have limited the accuracy of detailed anatomical descriptions.

Additionally, the comparison between pediatric and adult outcomes was descriptive only, lacking formal statistical testing due to the retrospective design.

Despite these limitations, this study provides valuable region-specific data that can inform targeted prevention strategies and clinical management in Northwest China.

### 4.8 Clinical and public health implications

Our findings have several practical implications. First, occupational safety measures in agricultural, manufacturing, and construction sectors should be strengthened, particularly in rural areas. Enterprises should provide appropriate personal protective equipment, including goggles, masks, helmets, and protective clothing. Second, child safety education should be prioritized in rural households, focusing on the safe storage of sharp objects and supervision of young children. Third, public awareness campaigns timed before the Lunar New Year should emphasize the dangers of fireworks and promote safe viewing practices. Fourth, healthcare systems should ensure timely access to specialized ophthalmic care for ocular trauma patients, particularly those in rural areas.

## 5 Conclusion

Ocular trauma in Northwest China predominantly affects male manual laborers in rural areas, with open-globe injuries accounting for the majority of cases. Pediatric trauma, primarily caused by sharp household objects, appears to carry a particularly poor prognosis based on descriptive observations, although formal statistical comparison with adults was not performed.

Injury zone, injury type (open vs. closed globe), and timing of presentation are independent prognostic factors for visual outcomes. Seasonal peaks during the Lunar New Year highlight opportunities for targeted public health interventions. These findings underscore the need for region-specific prevention strategies, including child safety education, workplace protection regulations, and holiday safety campaigns. Future multicenter studies with standardized follow-up protocols are needed to confirm these findings and guide evidence-based policy development.

## Data Availability

The original contributions presented in the study are included in the article/Supplementary Material, further inquiries can be directed to the corresponding author.
